# Millennia‐Long Evolution of Temperature and Salinity Dependence of a Baltic Sea Diatom Revealed by Resurrection Experiments

**DOI:** 10.1111/gcb.70408

**Published:** 2025-08-02

**Authors:** Sarah Bolius, Jana Hinners, Jérôme Kaiser, Silas Folgmann, Paula F. Steiner, Helge W. Arz, Anke Kremp

**Affiliations:** ^1^ Biological Oceanography Leibniz Institute for Baltic Sea Research Warnemünde Rostock Germany; ^2^ Helmholtz‐Zentrum Hereon Geesthacht Germany; ^3^ Marine Geology Leibniz Institute for Baltic Sea Research Warnemünde Rostock Germany

**Keywords:** adaptation, Baltic Sea, climate change, diatom, evolution, phytoplankton, resurrection, trait changes

## Abstract

Many species have demonstrated persistence through past climate phases, but the recent accelerated anthropogenic climate change severely impacts species composition, altering aquatic biodiversity and phytoplankton communities. Uncovering how species have responded to natural climate variability in the past is of great value for understanding adaptive dynamics and predicting future adaptations. Here, we investigated the Holocene adaptation dynamics of the cosmopolitan diatom species *Skeletonema marinoi* by reviving dormant phytoplankton cells that have accumulated in the sediment of the Baltic Sea. The Baltic Sea is strongly affected by current climate change and has undergone fundamental environmental changes throughout its Holocene history, including glacial rebound, alternating warmer and cooler periods, and changes in salinity and nutrient availability. Using resurrected temporal cohorts from up to 6800‐year‐old sediment horizons, we studied past adaptation dynamics by performing growth experiments and morphological measurements under different temperature and salinity conditions. Our results demonstrate that *S. marinoi* temporal cohorts exhibit differences in their morphological trait values and environmental optima, partially reflecting past ambient environments. Moreover, divergences from expected adaptation patterns demonstrate the complexity of evolution in natural ecosystems. Based on our findings, we expect *S. marinoi* to cope well with projected environmental changes for the Baltic Sea. These findings highlight the resilience of phytoplankton and emphasize their capacity for phenotypic adaptation to changing conditions. Furthermore, this research underscores the importance of understanding past adaptation processes in predicting phytoplankton responses to future climate change.

## Graphical abstract

Resurrected *Skeletonema marinoi* strains from Baltic Sea sediments, dating back up to 6800 years, revealed significant Holocene adaptation dynamics. Growth experiments under varied temperatures and salinities showed changes in environmental optima and morphological traits that partially reflect past conditions, although some divergences highlight evolutionary complexity. These findings emphasize the resilience and phenotypic adaptability of phytoplankton.

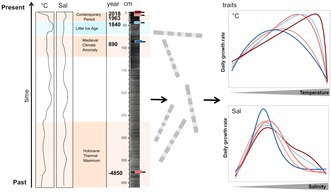

## 1 Introduction

We live in a time of increasing threats by anthropogenic environmental changes that are severely affecting ecosystems around the world (Thomas et al. 2004; Lodge et al. 2006; Grimm et al. 2013; Nolan et al. 2018; Priya et al. 2023). Much effort is therefore put into understanding how species respond to environmental changes (Walther et al. 2002; Pacifici et al. 2015; Fei et al. 2017; Nunez et al. 2019). Apart from migration and extinction (Pecl et al. 2017), possible responses include evolutionary adaptation to changing environmental conditions (Hendry et al. 2008; Irwin et al. 2015; Hinners et al. 2017). Most studies on species responses to environmental change use a ‘forward in tim’ approach based on for example, Intergovernmental Panel on Climate Change (IPCC) projections. They typically focus on one or two, sometimes several, altered environmental factors (Elena and Lenski 2003; Brennan et al. 2017). These approaches cannot take into account the complexity of real ecosystems, though. Most monitoring studies, in comparison, are time‐consuming and do not cover long‐enough time frames yet to assess long‐term adaptive changes in species.

Throughout Earth's history, the climate has alternated between warming and cooling, ocean salinity has fluctuated, and nutrient levels have varied (Bradley 2015). Some species have persisted through these different climate phases (Moritz and Agudo 2013). Yet, how they have responded to substantial climatic changes in the past remains largely unknown. Uncovering past adaptations will help to contextualize current environmental changes and enable more reliable model projections (Alsos et al. 2024; Hochfeld et al. 2025).

An approach to obtain information on species adaptation in the past is “resurrection”, in which dormant cells that are buried in the sediment are isolated and germinated (Kerfoot et al. 1999). Many plants, phytoplankton, zooplankton and microbial species produce resistant dormant cells to survive unfavourable conditions (Sallon et al. 2008; Pauwels et al. 2010; Ellegaard and Ribeiro 2018; Wörmer et al. 2019; Lennon et al. 2021). In aquatic environments, these dormant cells sink to the sediment, building up so‐called living “sediment archives” that preserve species information over space and time (Sundqvist et al. 2018; Lennon et al. 2021) and can remain viable for up to millennia (Legrand et al. 2019; Sanyal et al. 2022; Bolius et al. 2025). Preservation conditions are best in anoxic sediments with high sedimentation rates (Canfield 1994). Resurrecting dormant cells from distinct temporal cohorts allows to “travel in time” (Orsini et al. 2013), to reveal past adaptive changes and understand involved eco‐evolutionary processes.

So far, studies based on the resurrection approach have demonstrated evolutionary changes in various organism groups spanning up to 700 years, including adaptation to drought in plants (Johnson et al. 2022), to temperature in zooplankton and phytoplankton (Geerts et al. 2015; Medwed et al. 2024; Hattich et al. 2024), to eutrophication in zooplankton (Frisch et al. 2014), and co‐evolution of hosts and parasites in zooplankton (Decaestecker et al. 2007). Resurrection studies on phytoplankton that examine trait changes are rare and have mostly covered changes at the decadal scale (Hinners et al. 2017; Medwed et al. 2024; Hattich et al. 2024). A previous study in the Baltic Sea has demonstrated that phytoplankton species can endure much longer dormancy periods, though, even spanning thousands of years (Bolius et al. 2025). Using these millennia‐old cultures of the cosmopolitan diatom species *Skeletonema marinoi* revived from the Baltic Sea, we aim to track species evolution over time.

The Baltic Sea is a semi‐enclosed brackish water body, connected to the North Sea and very suitable for resurrection studies because of its seasonality and anoxic sediments. Due to its extremely strong anthropogenic influences, the Baltic Sea is also regarded as a ‘time machin’ for global climate change (Reusch et al. 2018). These anthropogenic influences include the rapid increase of the sea surface temperature (SST), with one of the fastest warming rates observed globally (Belkin 2009; Jamali et al. 2023) and an increasing number of heatwave events (Meier, Kniebusch, et al. 2022; Gröger et al. 2024; Lindenthal et al. 2024). Correspondingly, salinity is affected by ongoing climate change due to altered precipitation, ice melting, and ocean circulation (Meier, Kniebusch, et al. 2022), likely contributing to more pronounced salinity fluctuations. Further, notable changes in that is, timing and composition of phytoplankton have occurred within a few decades (Hjerne et al. 2019).

In order to study adaptation in the Baltic Sea beyond long‐term observations and instrumental data, paleoenvironmental reconstructions are available (Emeis et al. 2003; Zillén et al. 2008; Seppä et al. 2009; Andrén et al. 2011; Harff et al. 2011; Kabel et al. 2012; Kotrys et al. 2014; Warden et al. 2017; Kniebusch et al. 2019; van Helmond et al. 2020), making the Baltic Sea and its species a well‐suited study system. The Holocene history of the Baltic Sea since the Littorina transgression can be roughly divided into distinct climatic phases: the Holocene Thermal Maximum (HTM; 8000–4000 calibrated years Before Present; cal yr. BP with BP = 1950 Common Era, CE) with the warmest water temperature and salinity as high as today or higher; an intermediate phase with cooler temperature (4000–1000 cal yr. BP); the Medieval Climate Anomaly (MCA; 1000–700 cal yr. BP), a relatively warm period, but not as warm as the HTM, with relatively high salinity; the Little Ice Age (LIA; 700–150 cal yr. BP), the period with the coldest temperature and relatively low salinity; and the Contemporary Period (CP, since ca. 0 cal yr. BP), characterized by the ongoing fast increase in temperature and high fluctuations in salinity.

To better understand phytoplankton responses to changing climatic conditions and assess the future survival potential of these important primary producers, we analysed past and modern *S. marinoi* strains resurrected from sediment layers representing different Holocene climate regimes of the Baltic Sea history, reaching up to 6870 ± 140 years back in time (Bolius et al. 2025). We investigated whether and how *S. marinoi* strains isolated from five different time points of the Holocene are adapted to the respective environmental conditions. These strains cover three time points from the HTM (−4850 ce), the MCA (890 ce) and shortly after the LIA (1840 ce), and two from the CP (1963 and 2018 ce).

Our focus lay on temperature and salinity, two crucial abiotic environmental factors that affect phytoplankton (Kipriyanova et al. 2007; D'ors et al. 2016; Campbell et al. 2019) including *S. marinoi* (Judy et al. 2024) and also alternated substantially throughout the Holocene in the Baltic Sea (Emeis et al. 2003; Kabel et al. 2012; Kotrys et al. 2014; Warden et al. 2017; Wittenborn et al. 2022). Specifically, we analyzed cell properties and growth responses to temperature and salinity, and evaluated if the observed changes correlate with respective changes in ambient conditions. To better relate this to ambient past conditions, we also measured salinity values for the respective sediment horizons. Based on these results, we could assess the potential of *S. marinoi* to survive the progressive environmental changes of the next century including increasing temperature, a higher heatwave frequency, and changing salinities (Meier, Kniebusch, et al. 2022). Thereby, our study demonstrates that data from the past is invaluable for predicting future species responses.

## 2 Methods

### 2.1 Cultures of *Skeletonema marinoi*

The *Skeletonema marinoi* strains were resurrected from dated sediment cores (EMB262‐6‐28/30) of the Eastern Gotland Basin (EGB) as recently published in Bolius et al. (2025). The sediment cores were split in length, the surface carefully cleaned, the upper sediment discarded, and the samples were taken based on sedimentary ancient DNA protocols to avoid any contamination and sampling alterations (Epp et al. 2019).

The strains resurrected from the same sediment layer are referred to as “temporal cohort”, which is used as an alternative to populations since we cannot confidently classify them as populations. Temporal cohorts are from the years 2018 ± 2 ce (−68 ± 2 cal yr. BP), 1963 ± 5 ce (−13 ± 5 cal yr. BP), 1840 ± 15 ce (110 ± 15 cal yr. BP), 890 ± 110 ce (1060 ± 110 cal yr. BP), and −4850 ± 140 ce (6800 ± 140 cal yr. BP). For all experiments, we used 4 strains per temporal cohort. To simplify, in the following we will refer to the temporal cohort/strains in CE (without age uncertainties). All strains were cultured as clonal cultures at 4°C, 7.5 salinity, and with a 16:8 h light: dark cycle and 40 μmol photons m^−2^ s^−1^ photon fluence as stock cultures in f/2 medium with silicate (Guillard 1975) with sterile‐filtered (0.2 μm acetate cellulose filter) and autoclaved Baltic Sea water (7.5 salinity). These conditions represent standard conditions for recent cold‐adapted spring bloom phytoplankton and mimic the condition of the contemporary Central Baltic Sea (Naumann et al. 2024). We did not alter these conditions for different temporal cohorts.

### 2.2 Sea Surface Salinity Reconstruction

To obtain estimates for water surface salinity, sediments (ca. 1 g dry weight) from the same cores (EMB262‐6‐28/30) were extracted by accelerated solvent extraction (Thermo Scientific Dionex ASE 350) at high pressure and high temperature (100 bar and 100°C) with a mixture of dichloromethane and methanol (DCM:MeOH, 9:1, v:v). The total extracts were separated by microscale flash column chromatography using silica gel as solid phase into four fractions eluted respectively with *n*‐hexane, *n*‐hexane/DCM, DCM, and DCM/MeOH. The DCM fractions containing alkenones were analysed with a Trace1310 gas chromatograph (GC; Thermo Scientific) equipped with a split/splitless injector, a VF‐200 ms column (Agilent Technologies) and a flame ionization detector. Peak identification was based on comparing peak retention times with an in‐house sediment standard. C_37_ alkenone‐based sea surface salinity (SSS) estimates were obtained by applying the Baltic Sea calibration as published in Kaiser et al. (2017):

$$\mathrm{SSS}=\left(\%C_{37:4}–51.8028\right)/-5.8353$$

where %C_37:4_ = C_37:4_/(C_37:4_ + C_37:3_ + C_37:2_) * 100.

### 2.3 Water Temperature Reconstruction

For obtaining past subsurface water temperature conditions, we relied on published and unpublished estimates obtained with the TEX^L^_86_ paleothermometer (Schouten et al. 2003; Kim et al. 2010). In the Baltic Sea, the reconstructed temperatures refer to subsurface depths of 80–120 m, while surface temperatures are approximately 4°C warmer (Wittenborn et al. 2022). Cores from the Central Baltic Sea were used to estimate past water temperature: EMB201/7–4 (Kaiser et al. 2023), MSM51‐2/20 (Wittenborn et al. 2022), M86‐1/33–5, and 06EZ1215/11–2 (Dellwig et al. 2019). For the time horizons of the resurrected *S. marinoi*, the estimated subsurface temperature ranged between 3.0°C and 5.5°C (Table 1). Since the observed temperature variability remains consistent (Wittenborn et al. 2022), the derived temperature values can be considered relative to each other, indicating warmer or colder phases.

**TABLE 1:** Reconstructed subsurface temperature (error bar of ±0.3°C; Wittenborn et al. 2022) for the time horizons of the *Skeletonema marinoi* temporal cohorts.

Year (CE)	Reconstructed temperature (°C)	References
2018	5.5	(Kaiser et al. 2023)
1964	4.2	(Wittenborn et al. 2022)
1840	3.5	Kaiser, unpublished
890	3	Kaiser, unpublished
−4850	4.8	Kaiser, unpublished

Abbreviation: CE, common era.

### 2.4 Experimental Set‐Up

For temperature and salinity reaction norm measurements, the experiments were set up in the same way using 4 genetically different strains for each of the 5 investigated temporal cohorts (Bolius et al. 2025). The strains were analyzed by microsatellites, confirming that these are genetically distinct (Bolius et al. 2025). For temperature dependency analyses, experiments were conducted at 4°C, 7°C, 10°C, 13°C, 16°C, 20°C, 25°C, and 30°C. These experiments were set up with sterile‐filtered (0.2 μm acetate cellulose filter) and autoclaved Baltic Sea water (salinity 7.5) as base for f/2 medium with silicate (106 μM) (Guillard 1975). For salinity dependency analyses, salinity concentrations of 0.1, 2.5, 5, 7.5, 11, 15, 20, and 35 were tested, extending from fresh water to full marine salinity conditions. The salinity experiment was carried out at 4°C. This temperature was chosen as it represents the current spring conditions in the Central Baltic Sea and was also used for resurrection and cultivation (see above).

Autoclaved, sterile pure water was used as the medium base to allow for precise salinity manipulation. The water was supplemented as well with f/2 nutrients (Guillard 1975) and the salinity was adjusted via a sea salt and salinity optode measurement. Apart from the changes in temperature and salt conditions, the cultures were kept under control culturing conditions as described above.

To allow for a stepwise acclimation, clonal cultures of *S. marinoi* strains were inoculated during their exponential growth phase by adding 2 mL culture suspension to 40 mL f/2 medium (+ silicate 106 μM). The cultures were initially maintained under control culture conditions (temperature 4°C, salinity of 7.5). Then, the strains were acclimatized to the next temperature/salinity for always 6–7 days before moving to the next subsequent condition. This process was repeated under previous conditions for the next temperature/salinity until all strains reached their final experimental conditions (30°C, salinity of 2 and 35). Temperature always increased, while salinity either increased or decreased, starting from the original culturing conditions. This experimental design allowed all strains to acclimate slowly to experimental conditions, but meant that the experiment itself stretched over many weeks, during which the samples entered the experimental phase at different time points.

When the cultures reached exponential phase, the actual experimental phase was started. All starting cell concentrations were set up to a low concentration of cells (~3500 cells mL^−1^) based on triplicate chlorophyll‐*a* (chl‐*a*) fluorescence measurement (Plate Reader, infinite m plex Tecan, ext. 450 nm, em 682 nm). For each strain and temperature/salinity, 4 replicates to follow growth and 2 for cell size determination were set up in 24‐well plates (2 mL culture). The plates were closed with breathable plate‐seals (Breath‐Easy, Sigma Aldrich) to prevent any evaporation and make it possible to shake cultures before measurements. Growth was monitored every 1–3 days by chl‐*a* fluorescence measurement (plate reader) until the stationary phase was reached. The stationary phase was the phase without continuous exponential growth and was determined after 3 (measurement) days without further growth. During the mid‐exponential phase, 2 mL of the two replicates were fixed with Lugol's solution to examine the cell size from each strain and temperature/salinity. The mid‐exponential phase is defined as the midpoint between the start of exponential growth and the start of the stationary phase (no growth). After every chl‐*a* measurement, we checked the growth curve for mid‐exponential phase. In some cases, samples were taken on multiple days to ensure the right sample for the mid‐exponential phase was chosen. The growth rate per day (μ day^−1^) was calculated as average exponential growth for each strain for each temperature and salinity from the (visually estimated) start of the exponential growth to the start of the stationary phase, according to:

$$\mu \;{\mathrm{day}}^{-1}=\frac{\ln\left({\mathrm{RFU}}_{\mathrm{ty}}\right)-\ln\left({\mathrm{RFU}}_{\mathrm{tx}}\right)}{\mathrm{tx}-\mathrm{ty}}$$

RFU_ty_ represents the fluorescence of the culture at the end of the exponential phase, RFU_tx_ is the fluorescence of the culture at the beginning of the exponential phase, ty denotes the day at which the culture is at the end of the exponential phase, and tx the day of beginning of the exponential phase. The experiments ran at a 16:8 h light: dark cycle and 40 μmol photons m^−2^ s^−1^ photon light.

### 2.5 Morphological Traits

Length and width of *S. marinoi* were measured from the Lugol fixed cells in a mix of 2 replicates for one strain using an inverted light microscope (Axio Zeiss S100, Jena, Germany), camera (Olympus Lifescience, Tokio, Japan), and software (Olympus Stream Essential 2.4). At least 30 cells were measured. Based on these measurements, the cell biovolume for *S. marinoi* was calculated as a cylindrical cell (Hillebrand et al. 1999), as well as the width to length ratio. Likewise, cells per *S. marinoi* chain/filament were counted, at least 30 filaments.

### 2.6 Mathematical and Statistical Analyses

For temperature reaction norms, growth rate data for each strain at each temperature were fitted based on (Thomas et al. 2017) with the R package (R version 4.4.2) “rTPC” (version 1.0.4; model: thomas_2017; Padfield et al. 2021). From this fit, the optimum temperature (*T*_opt_) for each strain and temporal cohort was calculated. The salinity reaction norms were analysed using a generalized additive mixed model (GAMM) in the R package “mgcv” (version 1.9.1; Wood 2017). From these fits, the optimum salinity (Sal_opt_) was calculated for each strain and temporal cohort. As some strains only grew under one salinity condition, for example, 7.5 salinity (Figure S1) and thus no curve could be fitted, 8 strains had to be excluded from further salinity analysis.

As a measure of the width of the temperature/salinity niche, we calculated the temperature range within which 80% of the maximum growth rate was achieved. This was done by determining the maximum growth rate for each strain. The temperature window in which the growth rate exceeded 20% of the maximum growth rate was taken as the temperature niche.

All morphological traits (cell biovolume, width to length ratio, and cells per filament) were fitted using the GAMM function over the salinity or temperature gradient. As no growth was observed at 30°C, this treatment was excluded from morphological analysis.

To analyze the relation between past ambient environmental parameters and optimum temperature and salinity, we used a linear mixed‐effects model (as Optimum value ~ ambient environmental value * year + (1 | Strain)).

Statistical tests over reaction norms (Posthoc tests and pairwise comparisons) were performed in R using the “stats” (version 4.4.2) and “emmeans” packages (version 1.11.1; Searle et al. 1980; Lenth 2019). For the test of significance, the data were first tested for normal distribution by the Shapiro test, followed by ANOVA or Kruskal–Wallis test and suitable Posthoc tests using the base “stats” package in R.

As a proxy for variability, the coefficient of variation (CV) was calculated for each temporal cohort across *T*_opt_ and Salopt as:

$$\mathrm{CV}=\frac{\text{standard deviation}}{\text{mean}}$$

here based on data for temporal cohorts/years. The plasticity of strains as change in growth rate and morphological traits (cell biovolume, cells per chain) between culture conditions and under Sal_opt_ and *T*_opt_ conditions was calculated as relative change, based on Schaum et al. (2013):

$$\frac{\left(\mu_{\text{Xopt}}-\mu_{\mathrm{cc}}\right)}{\mu_{\mathrm{cc}}}$$

with *μ*_Xopt_ being the growth rate (or cell biovolume, cells per chain respectively) at Sal_opt_/*T*_opt_ and *μ*_cc_ growth (or cell biovolume, cells per chain, respectively) under control culture conditions, here 4°C and 7.5 salinity. The trait value at Sal_opt_/*T*_opt_ was matched with the predicted values from the model. To ascertain a match of predicted model values, a tolerance of 0.1 was included.

## 3 Results

### 3.1 Salinity Reconstruction

The %C_37:4_ water surface salinity estimates are based on lipids (C_37_ alkenones) produced by single‐celled phytoplankton (Isochrysidales) in the Baltic Sea (Kaiser et al. 2019). These salinity estimates varied between 6.8 and 8.2 (Table 2), with an uncertainty around 1 (Kaiser et al. 2017).

**TABLE 2:** Reconstructed surface salinity for the time horizons of the *Skeletonema marinoi* temporal cohorts.

Depth (cm)	Year (CE)	Surface salinity estimate
1	2018	8.2
20	1964	7.8
44	1840	6.8
106	890	7.3
382	−4850	7

Abbreviation: CE, common era.

### 3.2 Temperature Dependent Growth

**FIGURE 1:**
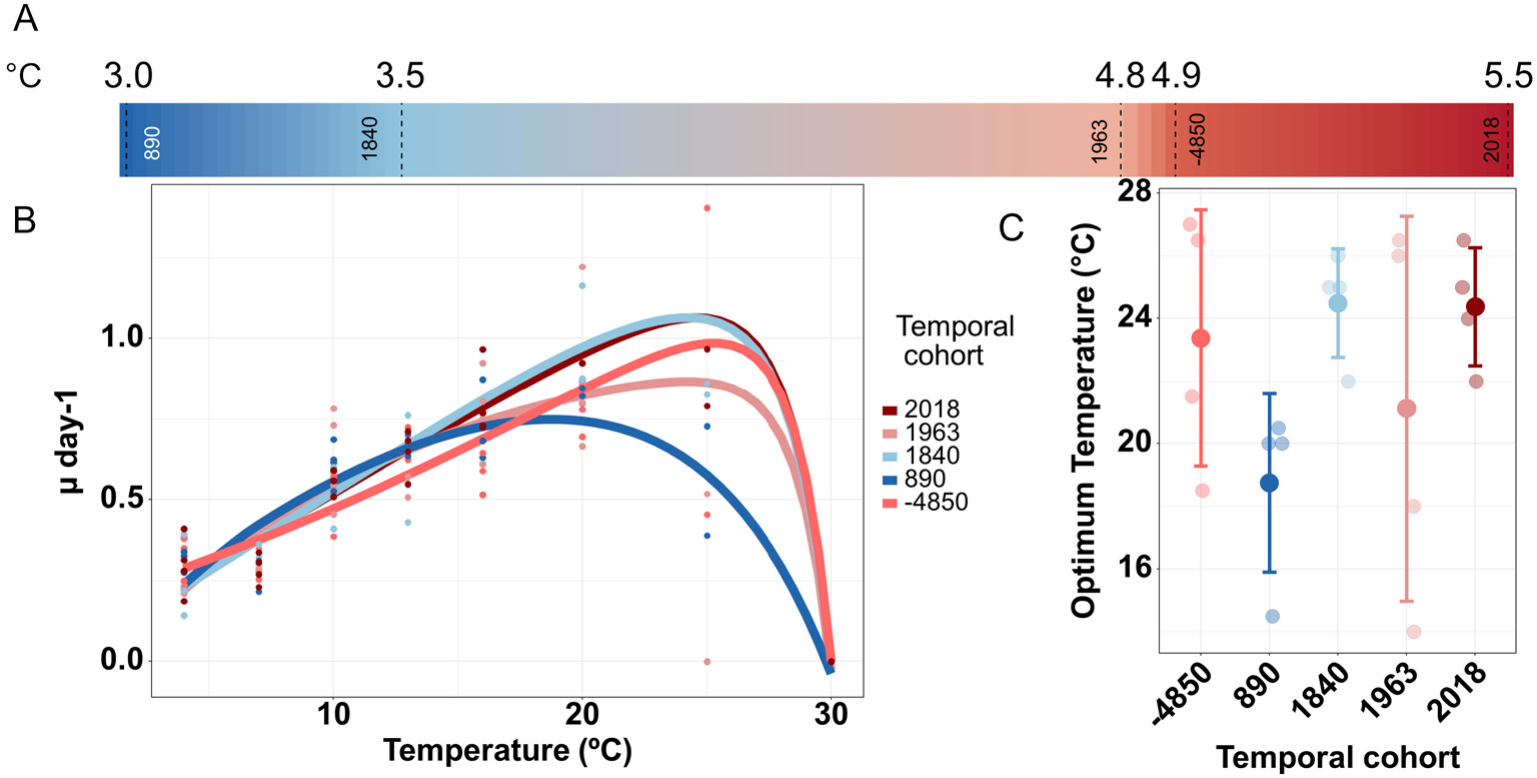
Reconstructed water temperatures and reaction norms for growth over temperature gradient. (A) Reconstructed Baltic Sea subsurface water temperature for the time horizons of the *Skeletonema marinoi* temporal cohorts. (B) Temperature‐dependent reaction norms for growth for each temporal cohort of *Skeletonema marinoi* by year. Fits are generated across all strains of a cohort. Note the overlapping solid lines for temporal cohorts 2018 and 1840 CE. (C) Optimum temperatures for each temporal cohort of *Skeletonema marinoi*, mean (solid point) and standard deviation (line) of the temporal cohorts, and mean values of individual strains (transparent points, based on 4 replicates per strain). The colors of lines and points in (B and C) refer to the reconstructed subsurface temperature values in (A).

The temperature reaction norms of the different temporal cohorts differed in shape, maximal growth rate (Figure 1B), and in optimum temperature (*T*_opt_, Figure 1C and Table 3). The critical thermal maximum was between 25°C and 30°C for all temporal cohorts, with no strain growing at 30°C (no temperatures tested between 25°C and 30°C). The temperature reaction norm dynamics did not differ significantly among temporal cohorts (GAMM Tukey Posthoc test: *p* > 0.335) and neither did the *T*_opt_ values (Kruskal–Wallis test, *p* = 0.2554). However, some qualitative differences in *T*_opt_ values are visible that appear to match the respective temperature conditions. While high *T*_opt_ values were observed in the most recent (24.4°C ± 1.89°C) and the oldest temporal cohorts (23.4°C ± 4.09°C), coinciding with warmer periods (5.5°C and 4.9°C subsurface water temperature), the lowest *T*_opt_ value (18.8 ± 2.84) was observed for the temporal cohort extracted from the coolest period (3.0°C). The variability in *T*_opt_ (measured as coefficient of variation, CV) differed significantly among temporal cohorts and ranged from 0.077 (1840‐temporal cohort) to 0.291 (1963 ce temporal cohort; Table 3). The width of the temperature niche, here defined as 80% of maximal growth rate, ranged from 8.88°C ± 3.64°C (−4850 ce temporal cohort) to 12.1°C ± 0.479°C (890 ce temporal cohort), including a temperature range of 8.5°C to 28.5°C (Table S1). The plasticity of temporal cohorts measured between growth rate at culturing conditions and *T*_opt_ showed substantial variability between strains, ranging from 1.55 ± 0.474 to 3.76 ± 2.54 (Table S1).

**TABLE 3:** Optimum temperature (*T*_opt_) and salinity (Sal_opt_), coefficient of variation (CV) of *T*_opt_ and Sal_opt_, and temperature and salinity niche width for growth, including minimum and maximum values, for resurrected temporal cohorts of *Skeletonema marinoi*.

Temporal cohort (year CE)	Temperature			Salinity		
	*T*_opt_ ± SD (°C)	CV of *T*_opt_	Niche width (min.—max.;°C)	Sal_opt_ ± SD	CV of Sal_opt_	Niche width (min.—max.)
2018	24.4 ± 1.89	0.077	9.5 ± 1.83 (14.5–28.5)	9.91 ± 1.76	0.178	7.21 ± 2.38 (5.88–16.45)
1963	21.1 ± 6.14	0.291	10.1 ± 3.42 (8.5–28.5)	7.90 ± 0.356	0.045	5.66 ± 0.18 (5.13–11.17)
1840	24.5 ± 1.73	0.071	9.88 ± 1.25 (16.5–28)	8.53 ± 0.889	0.104	7.04 ± 1.07 (4.88–13.93)
890	18.8 ± 2.84	0.152	12.1 ± 0.479 (9–25.5)	7.90 ± 0.356	0.045	4.02 ± 1.78 (5.63–10.92)
−4850	23.4 ± 4.09	0.175	8.88 ± 3.64 (11.5–28.5)	9.24 ± 0.809	0.088	6.16 ± 0.48 (5.63–13.68)

### 3.3 Salinity Dependent Growth

**FIGURE 2:**
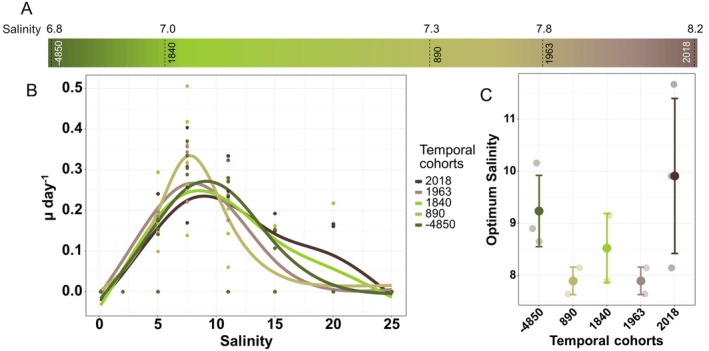
Reconstructed water salinity and reaction norms for growth over salinity gradient. (A) Reconstructed Baltic Sea surface salinity for the time horizons of the *Skeletonema marinoi* temporal cohorts. (B) Salinity‐dependent growth reaction norms for each temporal cohort by year. Fits are generated across all strains of a cohort (C) Optimum salinity for each temporal cohort, with mean (solid point) and standard deviation (line) of the temporal cohorts, and mean values of individual strains (transparent points, based on 4 replicates per strain). The colors of lines and points in (B and C) refer to the reconstructed surface salinity values in (A).

Salinity had a significant effect on growth (GAMM, *p* < 2e^−16^; Figure 2B,C). While none of the strains grew at the tested extreme salinities (low/freshwater: 0.1 and 2; high/marine: 25), all strains grew well at a salinity of 7.5 (Figure S1). The reaction norm dynamics over the salinity gradient did not differ significantly among temporal cohorts (pairwise comparison, *p* > 0.1529), but the optimum salinity (Sal_opt_) showed significant differences among temporal cohorts (Kruskal–Wallis test, *p* = 2.162e^−06^; Figure 2C and Table 3), partly following respective environmental salinity conditions. The Sal_opt_ of the oldest cohort (−4850 ce, 9.24 ± 0.809) has a significantly higher value than the Sal_opt_ of the subsequent cohorts (7.90 ± 0.356–8.53 ± 0.889 units) with lower corresponding environmental salinities (6.8–7.8). The most recent cohort (2018 ce) with its high Sal_opt_ is in accordance with the high environmental salinity of 8.2 determined at the time. The linear mixed‐effects model demonstrated a significant effect of year (*p* = 0.023) on Sal_opt_ and an interaction of year and ambient salinity (*p* = 0.025), but not of ambient salinity alone (*p* = 0.321). Sal_opt_ increased significantly with year (*p* = 0.025).

The width of the salinity niche ranged from 4.02 ± 1.78 to 7.21 ± 2.38, including salinities of 5.13 to 16.45 (Table S1). The variability among the temporal cohorts, measured as CV, varied for Sal_opt_ among 0.045 and 0.178 (Table S1). The plasticity of temporal cohorts measured between growth rate under culturing conditions and Sal_opt_, showing variability in trait values, differed among traits and temporal cohorts, ranging in between 0.181 ± 0.075 (−4850 ce temporal cohort) and 0.246 ± 0.076 (2018 ce temporal cohort; Table S1).

### 3.4 Effect of Temperature on Cell Morphology

**FIGURE 3:**
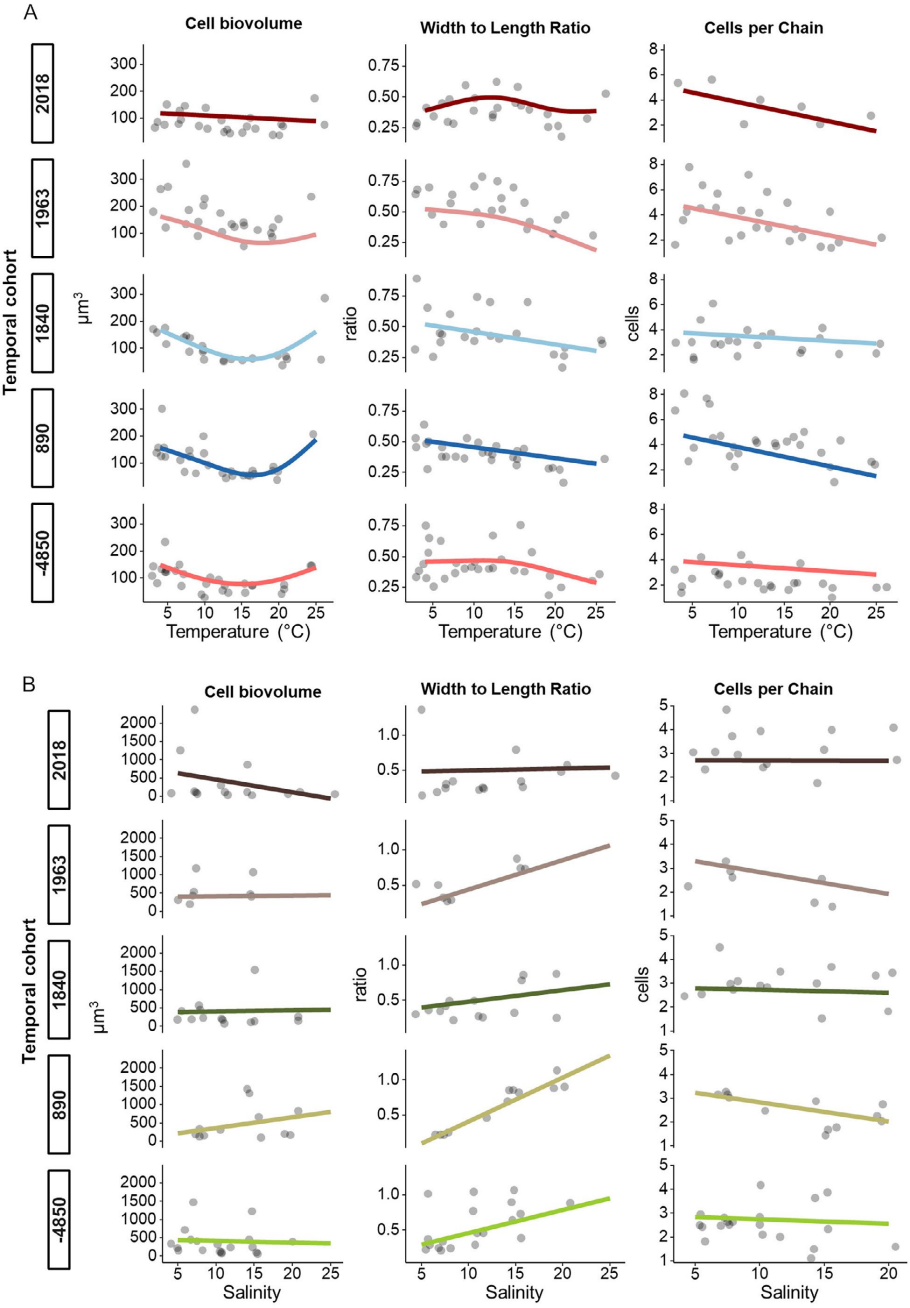
Morphological traits of the temporal cohorts of *Skeletonema marinoi* across the temperature (A) and salinity (B) gradients. Cell biovolume in μm^3^; width to length ratio of cells and cells per filament (left to right). The points represent mean values of each strain from the respective temporal cohort (year labelled on the left) and GAMM fit as line.

The temperature dependency of morphological traits showed substantial differences between cohorts. For all temporal cohorts, the cell biovolume decreased with increasing temperature up to 15°C–20°C, on average by 76% ± 54% (61 ± 40 μm^3^, Figure 3A) with a non‐linear relationship between temperature and cell biovolume (GAMM: *R* ^2^ = 0.572, *p* < 2e^−16^). The temporal cohort of 1963 ce had the highest decrease in cell biovolume along the temperature gradient (pairwise comparisons, significant differences compared to the temporal cohorts of 1840 ce, 890 ce and −4850 ce: *p* < 0.0028). The overall dynamics differed significantly between the temporal cohorts of 2018 ce, 1840 ce, and −4850 ce (GAMM Posthoc Test, *p* < 0.0482). At 15°C, the value of cell biovolume differed significantly among the temporal cohorts of 2018 ce, 1963 ce, 1840 ce, and 890 ce (GAMM pairwise comparison: *p* < 0.0176). The width‐toto‐length ratio changed by 42% ± 28% (0.17 ± 0.15 μm, Figure 3A) over the temperature gradient, mostly decreasing with higher temperature. The GAMM smooth term of cell biovolume over temperature was significant, except for the temporal cohort of 2018 ce (GAMM, *p* < 0.014492, 2018: *p* = 0.150187). The temporal cohort of 1963 ce differed significantly in their relationship of cell biovolume and salinity to the temporal cohorts of 2018 ce, 890 ce, and −4850 ce (GAMM Posthoc Test, *p* < 0.0195), with a higher decrease towards 25°C. The chain length, as cells per chain, decreased along the temperature gradient, but only significantly for the temporal cohorts of 2018 ce, 1963 ce, and 890 ce (GAMM smooth term, *p* > 4.68e^−05^), with no significant differences among years (Posthoc test, *p* > 0.1958) or at certain temperatures (Posthoc test, *p* > 0.261).

**FIGURE 4:**
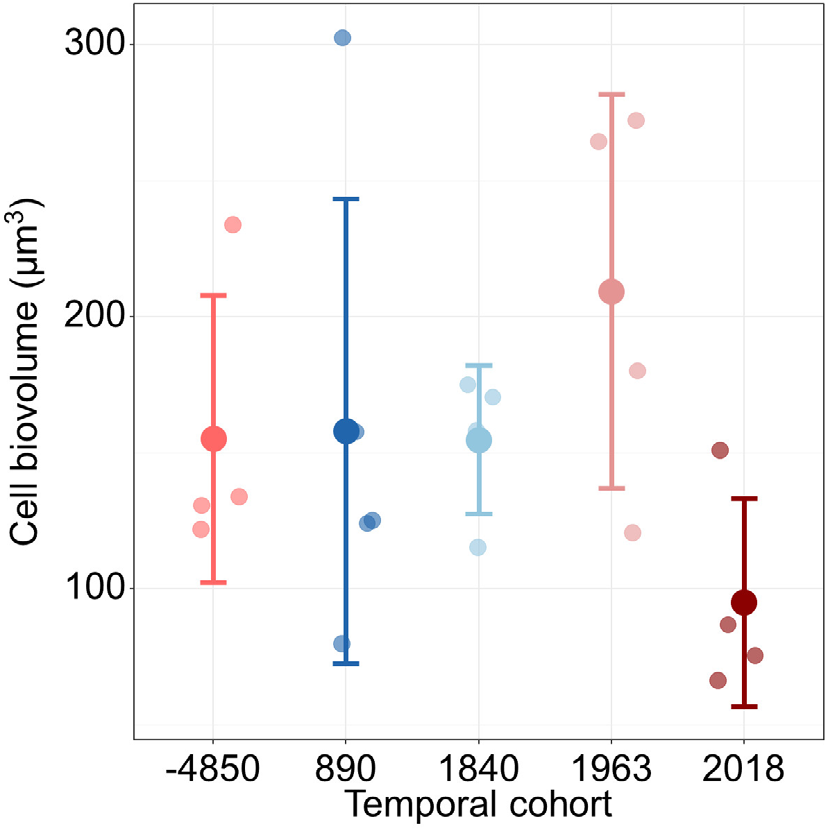
Mean cell biovolume of the temporal cohorts of *S. marinoi* at 4°C. Colors according to Figure 1.

When comparing the cell biovolume of temporal cohorts at 4°C (Figure 4), corresponding to the culturing condition, the contemporary Baltic Sea spring temperature (Naumann et al. 2024), and the temperature at which strains exhibit similar growth rates (Figure 1B), non‐significant differences were observed (Kruskal–Wallis test, *p* = 0.0695). The biovolume of the 1963 ce cohort shows the highest, whereas the recent temporal cohort shows the lowest biovolume.

### 3.5 Effect of Salinity on Cell Morphology

Similar to the temperature dependence, the salinity dependence of the morphological traits also shows large differences between the cohorts. The cell biovolume of the cells decreased or increased with salinity, depending on the temporal cohort (Figure 3B), but with no significant relationship to salinity (GAMM, *p* > 0.0794) or among temporal cohorts (Posthoc test, *p* = 1). Across the experimental salinity range, the cell biovolume changed across all temporal cohorts from 411 ± 368 μm^3^ at a salinity of 5 to 276 ± 242 μm^3^ at a salinity of 20 (only one value of 65 μm^3^ at salinity 25). The width to length ratio of *S. marinoi* cells was significantly related to salinity for temporal cohorts of 1963 ce, 890 ce, and −4850 ce (GAMM, *p* < 0.02680) and increased among salinity, except for the temporal cohort of 2018 ce. The chain length of temporal cohorts decreased with increasing salinity from on average 2.48 ± 0.366 at a salinity of 5 to 2.67 ± 0.830 at a salinity of 20, or remained unaffected with no overall significant relation between salinity and cells per chain (GAMM, *p* > 0.1443), except for the temporal cohort of 890 ce (GAMM, *p* = 0.0435). No significant differences between temporal cohorts (Posthoc test, *p* = 1) were found.

## 4 Discussion

In this study we were able to demonstrate changes in temperature and salinity dependence of growth and morphology of a Baltic Sea diatom over the past ~6,800 years of the Holocene. The identified temperature and salinity dependencies tend to partly reflect the respective environmental conditions of the five tested time horizons.

### 4.1 Temperature‐Dependent Growth Changes Across Time

Our data reveals a tendency of changes in the optimum growth temperature (*T*_opt_) over the tested temperature range among *S. marinoi* temporal cohorts from different time horizons throughout the Holocene. The cohorts from the two warmer phases, i.e., 2018 ce (Contemporary Period) and −4850 ce (Holocene Thermal Maximum), exhibit a higher mean *T*_opt_, whereas temporal cohorts from colder periods (with the exception of the 1840 ce temporal cohort) show an up to ~5°C lower *T*_opt_. These results suggest that *S. marinoi* adapts to respective ambient environments by adjusting temperature optima throughout time. The high optimum temperature of the cohort from just after the cold Little Ice Age period (1840 ce) demonstrates that also other biotic or abiotic factors may play a role in the adaptation to a certain temperature niche. One possible explanation could be a shifted bloom timing of *S. marinoi* from spring toward summer (Wasmund et al. 2008) due to altered competitive or grazing pressure. Alternatively, exceptionally warm years could have been captured. This could also be due to some reworking of older sediments deposited during warmer times, that is, non‐contemporaneous sediments, as described by Moros et al. (2020) for the Central Baltic Sea.

A increase in *T*_opt_ by ~3°C was observed in *S. marinoi* cohorts over the past 6 decades (temporal cohorts of 1963 ce and 2018 ce), during which the Baltic Sea has warmed by ~2°C (Baltic Marine Environment Protection Commission (Helsinki Commission—HELCOM) 2021). A recent study reported an increase of ca. 1°C in *T*_opt_ within the same time frame for coastal *S. marinoi* from the northern Baltic Sea (Hattich et al. 2024). Both examples demonstrate the strong capability of phytoplankton for rapid adaptation under natural conditions.

*T*_opt_ differences between temporal cohorts were not significant, likely due to high variability among strains, similar to results from other resurrection studies by Hinners et al. (2017) and Hattich et al. (2024). Such variability might be reinforced by seed banks, which integrate phenotypes from different seasons and years within a single temporal sediment layer. Moreover, our study location, the Eastern Gotland Basin, is a deep basin that collects and integrates resting stages over a large area due to its depth and currents (Hille et al. 2006). Currents, eddies, and Major Baltic Inflow events (Mohrholz 2018) in the Baltic Sea could transport particles like phytoplankton/resting cells (Bergen et al. 2018; Kudryavtseva et al. 2023; Paul et al. 2023). This also underscores the function of dormant cells and sediment archives as diversity reservoirs (Lundholm et al. 2011).

### 4.2 Temperature Dependency of Morphological Traits Across Time

The temperature dependency of morphological traits did not show large differences across temporal cohorts, indicating a stable response to temperature over millennia. From 4 to around 20°C, the cell biovolume of all *S. marinoi* strains decreased, in accordance with findings for other protists (Atkinson et al. 2003). The observed cell biovolume increase and the decreasing width‐to‐length ratio between 20°C and 25°C might be a stress response such as the building of sexual stages. The cells of *S. marinoi* elongate before sexual reproduction (Godhe et al. 2014), which goes along with an increase in cell size. However, we did not observe any further developed sexual stages like fertilized female gametes.

While changes in cell biovolume in response to temperature are similar across temporal cohorts, their cell biovolume at 4°C differs: it shows an increase for temporal cohorts until year 1963 ce, followed by a decrease for the 2018 ce temporal cohort (Figure 4). This cell size decrease aligns with Bergmann's Rule, with larger cells observed during colder phases (1963 ce, 1840 ce and 890 ce), and smaller cells during warmer phases (−4850 ce and 2018 ce temporal cohorts). Interestingly, the 1840 ce temporal cohort also shows a larger cell biovolume compared to the 2018 ce temporal cohort (rather cold adaptation) demonstrating again that adaptation may be influenced by multiple biotic and abiotic factors. This was recently demonstrated genetically for *S. marinoi* (Pinseel et al. 2025).

The decrease in chain length with higher temperatures aligns with previous findings for *S. marinoi* (Li et al. 2021) and a reduced proportion of chain‐forming species under increased temperature (Sugie et al. 2020). Strains from the Holocene Thermal Maximum (i.e., the −4850 ce temporal cohort) generally exhibited fewer cells per chain compared to the other cohorts, maybe due to their origin under longer‐lasting warmer climate conditions.

### 4.3 Salinity‐Dependent Growth Changes Across Time

Our results reveal significant changes in salinity optima ranging between 7.9 and 9.9 salinity units. Based on our surface salinity estimates, the salinity ranged between 6.8 and 8.2 over the 5 time points of the last 6800 years. As these reconstructions are for surface waters, they may not fully account for all salinity changes throughout the water column which can also explain some unexpected adaptation patterns. While our derived surface salinity for −4850 ce is comparatively low, we observed one of the highest Sal_opt_ for the respective temporal cohort. Another study, however, derived a comparatively higher salinity for this time horizon (Kotrys et al. 2014), indicating a potential measurement inaccuracy in our analysis. The high Sal_opt_ of the oldest cohort may, however, also be explained by the potential origin of *S. marinoi*, which we discuss in more detail further below. The salinity niche of all temporal cohorts is restricted to brackish conditions, demonstrating their strong local adaptation to the conditions of the Baltic Sea (Sjöqvist et al. 2015). However, other studies demonstrate way broader salinity tolerances for *S. marinoi* (Sjöqvist 2015; Pinseel et al. 2022; Orizar and Lewandowska 2022). As explained previously with regard to temperature, the deep Eastern Gotland Basin stores resting stages over a large area, over which the environmental salinity may vary. Based on our salinity trait data, we can conclude that the strains likely originated from the central to southern Baltic Sea, where salinities range between 8 and 10.

While increases in temperature in temperate and polar aquatic habitats are usually associated with decreased salinity due to ice melting and river run‐offs (Morley et al. 2020), our results suggest the contrary, i.e., higher salinity optima for strains from warmer periods (strong positive correlation of *T*_opt_ and Sal_opt_—Spearman rank, rho = 0.593, *p* = 0.092). In line with our findings, other model‐based environmental reconstructions reported the Holocene Thermal Maximum to be a period where high temperature and salinity co‐occurred in the Baltic Sea, though (Andrén et al. 2011).

### 4.4 Salinity Dependency of Morphological Traits Across Time

We observed minimal changes in morphological traits across the salinity gradient and among the temporal cohorts. While higher salinity has been previously reported to induce sexual reproduction as well as thereby the elongation of cells (Godhe et al. 2014), this has not been observed here. In contrast, all strains have a higher width to length ratio (=shorter cells) under higher salinities.

### 4.5 Nutrient Regimes Across Time

A recent study from the northern Baltic Sea (Hattich et al. 2024) demonstrated higher growth rates in strains exposed to higher eutrophication, attributing this to nutrient‐repleted conditions. Similarly, we observed high maximum growth rates for the oldest cohort (−4850 ce) and most recent (2018 ce), which align with the assumed increased nutrient concentrations together with higher temperatures during the Holocene Thermal Maximum (van Wirdum et al. 2019) and today. The growth rate for the temporal cohort of 1963 ce was comparatively lower, although this decade was also marked by higher nutrient inputs (Andersen et al. 2017; Murray et al. 2019). Nutrient conditions cannot only lead to increased growth, but also larger cell sizes though (Irwin et al. 2006). The significantly larger cell biovolume of the 1963 ce temporal cohort, as well as the slightly larger cell biovolume from the oldest cohort than the 2018 ce temporal cohort (Figure 4) might thus be explained by higher nutrient concentrations during these times.

### 4.6 Shortcomings of This Study

While substantial effort was invested in resurrecting as many strains as possible from each sediment layer, the germination success was relatively low (Bolius et al. 2025), resulting in a low number of viable strains from each cohort. This limitation reduced the statistical power of the study. Additionally, the study could not account for other biotic and abiotic factors, such as nutrient availability, grazing pressure, and competition, which could have influenced adaptive dynamics, but were not resolved within the study's scope.

The initial selection of strains and traits could have been influenced by the laboratory conditions during resurrection. As all cultured strains showed a higher *T*_opt_ than the culturing conditions of 4°C, we believe it is unlikely that the temperature conditions during resurrection introduced a bias. For salinity, though, some strains showed a very low salinity tolerance which might be associated with the culturing conditions.

Moreover, potential cross‐dependencies between temperature and salinity were not investigated, which could have provided further insights into the evolution in a complex natural environment.

### 4.7 Potential Origin of *S. Marinoi* and Possible Implications for the Future

The *T*_opt_ of the resurrected *S. marinoi* strains is substantially higher than the mean temperature of the Baltic Sea (Naumann et al. 2024), especially during spring, when *S. marinoi* mainly occurs and produces high biomasses (Zettler et al. 2024). The growth rate optimum of phytoplankton generally lies above the ambient temperature (Thomas et al. 2012). Still, the high temperature optimum of *S. marinoi* and its ability to thrive in warm water (Sarno et al. 2005; Zingone et al. 2005) suggest that *S. marinoi* could have originated from warmer waters. The majority of the marine Baltic Sea species originated from the North Sea and North Atlantic regions (Segerstråle 1957; Kautsky and Svensson 2003) when the Baltic Sea became connected to these waters due to the sea level rise (Andrén et al. 2011). The deposition period of the oldest cohort from −4850 ce is shortly after the connection of the Baltic Sea with the open ocean. The high *T*_opt_ of the oldest cohort from −4850 ce could thus not only mirror environmental conditions at the time, but also indicate that these strains might have “just” invaded the Baltic Sea from warmer waters. Also, the significantly higher Sal_opt_ of the oldest cohort supports this hypothesis. However, the strong local adaptation to brackish conditions and the lacking ability of all cohorts, including the oldest, to grow at high salinities contradict this theory.

The ongoing Contemporary Period is associated with a rapid temperature increase and fluctuating conditions like heat waves (Meier, Kniebusch, et al. 2022; Meier, Dieterich, et al. 2022; Lindenthal et al. 2024). This may have led to the increase in plasticity and thermal niche width, which we observed in the most recent cohort compared to the oldest cohort (Tables 2). These differences might be caused by the longer, more stable conditions during the Holocene Thermal Maximum in comparison to the current rapid increase in temperature. According to current IPCC scenarios, the Baltic Sea is projected to warm by 3°C over the next century (Intergovernmental Panel on Climate Change 2023), and heatwaves are projected to increase in occurrence, intensity, and duration (Meier, Kniebusch, et al. 2022; Meier, Dieterich, et al. 2022; Gröger et al. 2024), leading to more fluctuating conditions. Our data suggest that even under such elevated temperatures and more frequent and intense heat waves, *S. marinoi* is likely to continue to thrive. Future model projections that consider evolutionary adaptation for example (Hinners et al. 2019; Hochfeld and Hinners 2024), can make use of our data to further explore how *Skeletonema marinoi* may respond to the ongoing climate change.

## 5 Conclusion

In this study, we investigated the temperature and salinity dependence of a diatom resurrected from up to 6800‐year‐old sedimentary archives of the central Baltic Sea. To our best knowledge, this is the longest time span ever investigated in a phytoplankton resurrection study. Our data revealed adaptations to temperature and salinity regimes that mostly, but not always, matched the respective environmental conditions. These findings do not only demonstrate the potential of phytoplankton to adapt to environmental conditions, but also the complexity of real ecosystems, sometimes causing unexpected adaptations.

Our findings provide further insights into and support to the notion of the “adaptation history” of species. Since some of the investigated climate phases were as warm as today, understanding past adaptations offers conclusions to be drawn about the ongoing adaptation to climate change and provides a baseline for species adaptation in diatoms over the past ~7,000 years. The comparison of modern strains of *S. marinoi* with strains of the Holocene Thermal Maximum suggests that modern *S. marinoi* is not at its thermal limits yet and indicates a resilience to climatic changes.

Our millennia‐spanning results highlight the power of resurrection studies not only for studying the past, but also for learning about the future and emphasize the role of sediment archives as reservoirs of biodiversity. Tracking and understanding past trait changes of species in response to environmental conditions is crucial, especially with the rapid climatic changes currently observed.

## Author Contributions

**Sarah Bolius:** conceptualization, data curation, formal analysis, investigation, methodology, validation, visualization, writing – original draft, writing – review and editing. **Jana Hinners:** formal analysis, validation, visualization, writing – review and editing. **Jérôme Kaiser:** investigation, methodology, resources, software, validation, writing – review and editing. **Silas Folgmann:** investigation, methodology, writing – review and editing. **Paula F. Steiner:** investigation, methodology, writing – review and editing. **Helge W. Arz:** investigation, methodology, resources, validation, writing – review and editing. **Anke Kremp:** conceptualization, funding acquisition, project administration, resources, supervision, validation, writing – review and editing.

## Conflicts of Interest

The authors declare no conflicts of interest.

## Supporting information


**Data S1:** gcb70408‐sup‐0001‐supinfo.pdf.

## Data Availability

The data that support the findings of this study are openly available on the Leibniz Institute for Baltic Sea Research's repository at https://doi.io‐warnemuende.de/10.12754/data‐2025‐0007.
