# Supplementary material for: Millennia‐Long Evolution of Temperature and Salinity Dependence of a Baltic Sea Diatom Revealed by Resurrection Experiments
Source: Glob Chang Biol. 2025 Aug 2;31(8):e70408. doi: 10.1111/gcb.70408 (PMC12317337; doi:10.1111/gcb.70408)
Supplement: Supplementary file 1 — Data S1: gcb70408‐sup‐0001‐supinfo.pdf. [file GCB-31-e70408-s001.pdf]

Supplementary Information

Millennia-long evolution of temperature and salinity dependence of a Baltic Sea diatom revealed by resurrection experiments

Sarah Bolius[1], Jana Hinners[2], Silas Folgmann[1], Paula F. Steiner[1], Jérôme Kaiser[3], Helge W. Arz[3], Anke Kremp[1]

[1] Biological Oceanography, Leibniz Institute for Baltic Sea Research Warnemünde, Rostock, 18119 Germany

[2] Helmholtz-Zentrum Hereon, Geesthacht, 21502 Germany

[3] Marine Geology, Leibniz Institute for Baltic Sea Research Warnemünde, Rostock, 18119 Germany

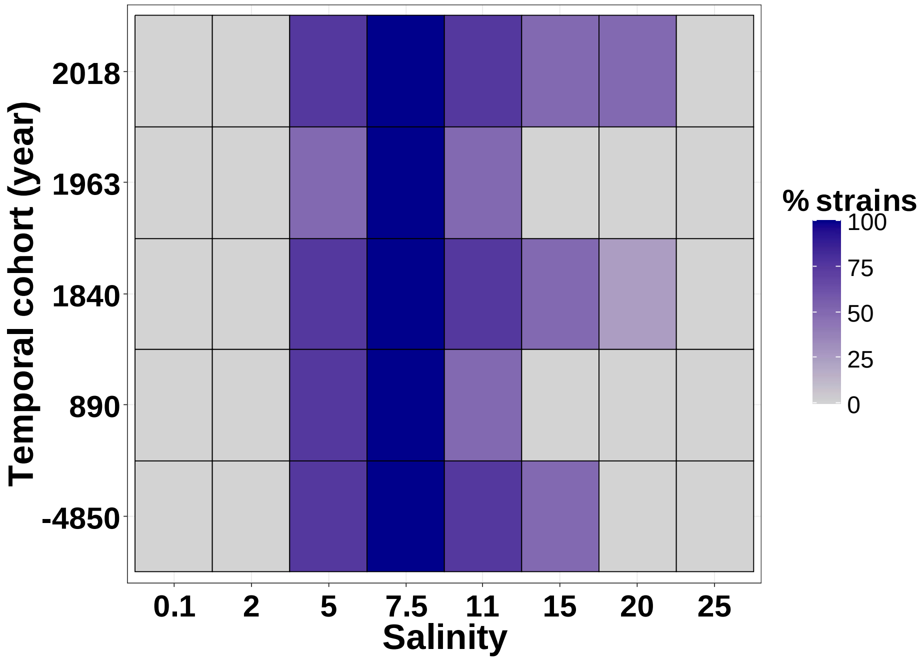

Supplementary Information Fig S1: Heatmap of strain percentage per temporal cohort that grew over tested salinities.

Supplementary Information Table S1: Plasticity values (absolute numbers) for temporal cohorts in shift of growth rate, cell biovolume, and cells per chain between culturing and optimal temperature and salinity conditions.

	**Temperature**			**Salinity**		
**Temporal cohort (year)**	**Growth rate**	**Cell biovolume**	**Cells per chain**	**Growth rate**	**Cell biovolume**	**Cells per chain**
2018	3.08 ± 1.83	0.163 ± 0.149	0.362 ± 0.043	0.246 ± 0.076	2.531 ± 1.794	0.154 ± 0.104
1963	3.36 ± 2.45	0.500 ± 0.105	0.551 ± 0.719	0.227 ± 0.025	0.403 ± 0.397	0.082 ± 0.008
1840	3.76 ± 2.54	0.235 ± 0.213	0.626 ± 0.488	0.237 ± 0.204	0.842 ± 0.264	0.200 ± 0.181
890	1.55 ± 0.474	0.468 ± 0.115	0.357 ± 0.218	0.232 ± 0.038	1.695 ± 0.399	0.144 ± 0.006
-4850	2.79 ± 1.90	0.537 ± 0.140	0.888 ± 0.768	0.181 ± 0.075	0.747 ± 0.606	0.066 ± 0.051
